# Profiling chimeric RNA in prostate cancer in Chinese cohorts reveals similarities and differences compared to Western populations

**DOI:** 10.1002/imt2.70014

**Published:** 2025-03-13

**Authors:** Qiong Wang, Shunli Yu, Jirong Jie, Justin Elfman, Zhi Xiong, Sandeep Singh, Samir Lalani, Yiwei Wang, Kaiwen Li, Bisheng Cheng, Ze Gao, Xu Gao, Hui Li, Hai Huang

**Affiliations:** ^1^ Department of Urology, Nanfang Hospital Southern Medical University Guangzhou China; ^2^ Department of Pathology, School of Medicine University of Virginia Charlottesville Virginia USA; ^3^ Department of Urology, Sun Yat‐sen Memorial Hospital Sun Yat‐sen University Guangzhou China; ^4^ Guangdong Provincial Key Laboratory of Malignant Tumor Epigenetics and Gene Regulation, Sun Yat‐Sen Memorial Hospital Sun Yat‐Sen University Guangzhou China; ^5^ Department of Urology, Changhai Hospital Naval Medical University Shanghai China; ^6^ Department of Urology The Sixth Affiliated Hospital of Guangzhou Medical University, Qingyuan People's Hospital Qingyuan China

## Abstract

Chimeric RNAs from chromosomal rearrangements have long been validated as cancer markers and therapeutic targets for many years. Recently, trans‐splicing and cis‐splicing between adjacent genes are also shown to generate chimeric RNAs. They influence tumor progression by coding fusion proteins, acting as long noncoding or circular RNAs, or altering parental gene expression. Here, we analyzed chimeric RNAs from The Cancer Genome Atlas and Chinese Prostate Cancer Genome and Epigenome Atlas, identifying similarities and differences between Western and Chinese prostate cancer (PCa) cohorts. We confirmed distinct chimeric RNA expression patterns among cancer epithelial cells, cancer‐associated fibroblasts, tumor‐associated macrophages, and T cells. We unraveled how these chimeras impact tumor cell growth, stromal cell transformation, and intercellular communication within the microenvironment. This comprehensive study establishes a chimeric transcriptome atlas for Chinese PCa patients, highlights population‐specific disparities, and presents validated chimeric RNAs with diagnostic, prognostic, and therapeutic potential.

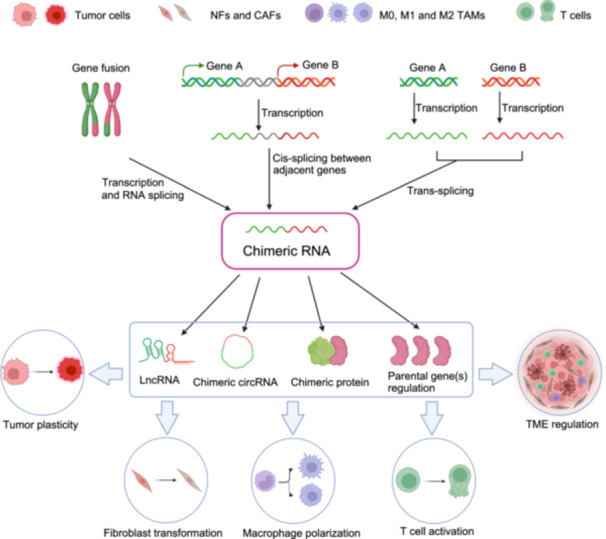

To the Editor,

Prostate cancer (PCa) is the most common malignant tumor in men and exhibits significant racial disparities. Incidence rates vary widely, with the highest recorded in Northern Europe and lowest in South Central Asia [1]. Conversely, the Asia population has the highest mortality‐to‐incidence rate ratio (MR/IR) [2]. Genomes and transcriptomes from large Western clinical datasets have been comprehensively studied. However, the characteristics of PCa in Western populations may not fully apply to Chinese populations.

Chimeric RNAs have been validated as cancer diagnostic and therapeutic targets for many years and can influence tumor progression through various mechanisms [3, 4, 5, 6]. The landscape of chimeric RNAs in several cancers have been characterized, but they primarily rely on immortalized tumor cells, which inevitably neglects other crucial cell types within the tumor microenvironment (TME). Additionally, comprehensive landscape research on PCa‐associated chimeric RNAs is still lacking, particularly in Chinese populations.

Here, we utilized chimeric RNAs from The Cancer Genome Atlas (TCGA) and Chinese Prostate Cancer Genome and Epigenome Atlas (CPGEA), and identified similarities and differences between the two cohorts. We validated numerous chimeras that showed differential expression between tumors and adjacent normal tissues. Importantly, these chimeras are distributed with varying frequencies across tumor and surrounding cells, including cancer‐associated fibroblasts (CAFs), tumor‐associated macrophages (TAMs), and T cells. The racial differences in chimeric RNA highlight the need for detailed PCa studies across diverse populations. The large number of validated functional chimeric RNAs providing a list of potential biomarkers and/or therapeutic targets in Chinese populations.

## RESULTS AND DISCUSSION

### Discovery pipeline and characterization of chimeric RNAs

**Figure 1:**
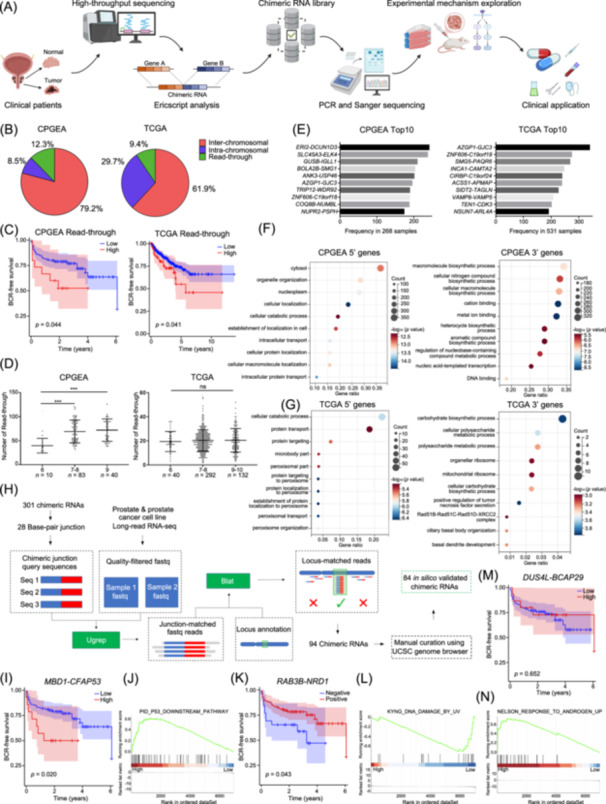
Discovery, identification, and classification of chimeric RNAs. (A) The pipeline for the discovery, validation, functional analysis, and clinical application of chimeric RNAs. (B) A total of 30,315 non‐M/M chimeric RNAs were categorized based on their fusion types in Chinese Prostate Cancer Genome and Epigenome Atlas (CPGEA), and 11,749 in The Cancer Genome Atlas (TCGA). (C) BCR‐free survival analyses based on the number of read‐through chimeric RNAs in CPGEA and TCGA database (87.5% low vs. 12.5% high). (D) The number of read‐through chimeric RNAs was significantly lower in Gleason score 6 patients in CPGEA, with no difference among different Gleason score tumor samples in TCGA. (E) The top 10 most frequently occurring chimeric RNAs in CPGEA and TCGA. (F, G) Gene ontology analyses of the 5′ and 3′ parental genes involved in the identified chimeric RNAs in CPGEA and TCGA. (H) UGREP pipeline diagram illustrating in silico long‐read validation approach. (I, J) A representative Type I chimeric RNA, *MBD1‐CFAP53* was correlated with poor prognosis. GSEA differential gene analysis based on the read counts of *MBD1‐CFAP53*, showing enrichment in the P53 pathway. (K, L) A representative Type II chimeric RNA, *RAB3B‐NRD1* was correlated with good prognosis. Gene Set Enrichment Analysis (GSEA) differential gene analysis based on the read counts of *RAB3B‐NRD1*, showing enrichment in DNA damage‐related pathways. (M, N) A representative Type III chimeric RNA, *DUS4L‐BCAP29* showed no correlation with prognosis. GSEA differential gene analysis based on the read counts of *DUS4L‐BCAP29*, showing enrichment in androgen response‐related pathways. ***p < 0.001.

After Ericscript prediction [7, 8], 25,944 chimeric RNAs were predicted in TCGA and 101,973 in CPGEA (Figure 1A and Tables S1, 2). They can be categorized into E/E, E/M, M/E, and M/M based on junction site [9]. We filtered out the M/M‐type transcripts because of their lower validation rate [10], leaving 30,315 chimeras in CPGEA and 11,749 in TCGA. The difference in the total number of chimeras is presumably due to the difference in sequencing depth. We further divided the chimeric RNAs into read‐through, inter‐chromosomal and intra‐chromosomal based on the location of the chromosomes [9]. Inter‐chromosomal chimeras are the most frequent type, presumably the products of chromosomal rearrangement or trans‐splicing, suggesting these two mechanisms are major contributors for chimeric RNA (Figure 1B).

Different from inter‐chromosomal and intra‐chromosomal (Figure S1A,B), we found that more read‐through chimeras predicted worse prognoses (Figure 1C). Notably, read‐through chimeras are significantly higher in cancer tissues than adjacent noncancerous tissues in TCGA (Figure S1C), but not in CPGEA (Figure S1D). In CPGEA, high Gleason score tumors show more read‐through chimeras than low‐score tumors, with no difference in TCGA (Figure 1D). These findings suggest read‐through chimeras are linked to tumor occurrence in Western cohorts but to tumor progression in the Chinese cohort.

We focused on recurrent chimeric RNAs in ≥5 patients. After BLAT validation, 384 were identified in TCGA and 1351 in CPGEA (Figure S1E). We listed the top ten chimeras with the highest expression frequencies (Figure 1E). Several frequent chimeric RNAs differ between the two cohorts, suggesting that certain chimeric RNAs may be related to PCa disparities and can guide ethnicity‐specific diagnostics and therapies.

Gene ontology (GO) analysis of parental genes showed that 5′ genes in both TCGA and CPGEA were enriched in protein processing, degradation, and transport. For 3′ genes, TCGA showed enrichment in glucose metabolism, while CPGEA showed macromolecular biosynthesis, nitrogen compound biosynthesis, and cation binding (Figure 1F,G). These results suggest 5′ genes are involved in shared pathways, while 3′ genes may regulate PCa progression, possibly linked to higher MR/IR in Chinese cases.

### The validation of chimeric RNAs in the Chinese population

Due to limited research and poorer prognosis, we focused on Chinese PCa‐related chimeric RNAs. About half of the 1351 chimeras had their 3′ gene breakpoints located in the 3′ UTR, but none were validated by Reverse Transcription Quantitative Polymerase Chain Reaction (RT‐qPCR), indicating a high false‐positive rate for this type of chimeric RNA (data not shown). We then focused on the remaining 635 chimeras (Figure S1F), with 301 showing differential expression between tumor and normal tissues after AGREP analysis (Table S3). We further performed a novel pipeline for in silico long‐read validation and found high‐confidence long‐read support for 84 of them (Figure 1H and Tables S4, 5) [11].

Primers were designed annealing to the parental genes and flanking the junction site (Table S6). Mixed cDNAs from 34 pairs of clinical PCa samples were used for RT‐qPCR, and 101 chimeric RNAs were confirmed by Sanger sequencing, most of which were novel (Figures S1F, 2, 3). We noticed a significant overlap between the experimental and in silico validated chimeras (Figure S4A), confirming the high accuracy of our newly developed pipeline.

We categorized the 101 chimeric RNAs into three types. Type I, higher expression levels in tumor tissue are associated with worse prognosis. For instance, *MBD1‐CFAP53* is elevated in PCa (Figure S4B) and correlates with worse outcomes (Figure 1I). Differential expression analysis of *MBD1‐CFAP53* showed enrichment in the P53 downstream pathway (Figure 1J, Figure S4C), a key regulator of PCa progression [12]. Type II, higher expression levels in tumor tissue are associated with better prognosis, suggesting a potential relationship with treatment sensitivity, similar to the case of *NEIL3* we reported previously [13]. For instance, *RAB3B‐NRD1* is highly expressed in tumors (Figure S4D) but associated with better outcomes (Figure 1K). Differential expression analysis revealed enrichment in the DNA damage‐related pathway (Figure 1L, Figure S4E), suggesting that *RAB3B‐NRD1* may share a similar function with *NEIL3*. Type I and II chimeras, therefore, represent a hidden repertoire of novel prognostic biomarkers and/or therapeutic targets. Type III, the expression of chimeric RNAs shows no correlation with prognosis. Nearly all PCa patients initially receive androgen deprivation therapy (ADT); if these chimeras are involved in androgen receptor (AR)‐associated pathways, ADT treatment may mask their effects on PCa progression. For instance, *DUS4L‐BCAP29* is overexpressed in tumors (Figure S4F) but shows no correlation with prognosis, and differential expression analysis revealed enrichment of the AR response pathway (Figure 1M, N, Figure S4G).

### Expression of chimeric RNAs in tumor cells and TME

**Figure 2:**
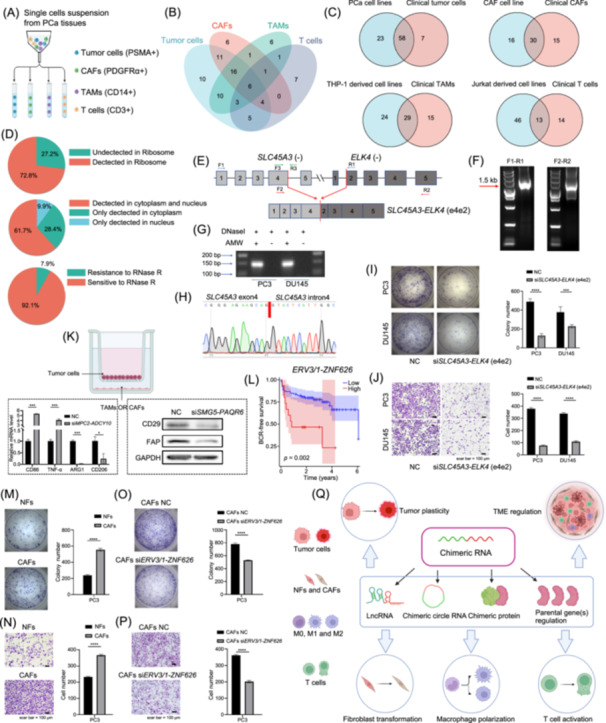
Distribution of chimeric RNAs in tumor and stromal cells and their functional roles. (A) Schematic diagram illustrating the isolation of cancer cells, cancer‐associated fibroblasts (CAFs), tumor‐associated macrophages (TAMs), and T cells from clinical samples using FACS. (B) Venn diagram showing the overlap of chimeric RNAs among clinical cancer cells, CAFs, TAMs, and T cells. (C) Comparison of chimeric RNA intersections between clinical cancer cells and PCa cell lines, clinical CAFs and immortalized CAF cell lines, clinical TAMs, and TAM‐related cell lines, as well as clinical T cells and T cell lines. (D) The classifications of chimeric RNAs based on whether they can be detected in the ribosome, their distribution in the nucleus and cytoplasm, and whether they are resistant to RNase R. (E) Schematic diagram of read‐through event between *SLC45A3* and *ELK4*. Blocks represent exons, lines represent introns or intergenic regions, and red arrows indicate junction sites. Primers were designed based on exonic or intronic regions of parental genes. (F) Gel images of Touch‐down polymerase chain reaction (PCR) products showing the full length of *SLC45A3‐ELK4* (e4e2) using two different primer pairs. (G) RNAs from cancer cell lines were treated with DNaseI and separated into two groups: with or without Avian Myeloblastosis Virus Reverse Transcriptase (AMV RT). Correct products were observed only in samples with AMV RT. (H) Sanger sequencing validated the junction sequence between exon 4 and intron 4 of *SLC45A3*. (I) Colony formation assay to assess cell viability in PC3 and DU145 cells following knockdown of *SLC45A3‐ELK4* (e4e2). (J) Representative images and histograms of migration assays in PC3 and DU145 cells after knockdown of *SLC45A3‐ELK4* (e4e2). (K) Coculture system of PC3 cells with TAMs or CAFs. Left panel: RT‐qPCR analysis of M1‐ and M2‐related marker expression levels in M0‐phenotype TAMs cocultured with PC3 cells following *MPC2‐ADCY10* knockdown. Right panel: Western blot analysis of changes in CAF‐related markers after coculture of CAF cell lines with PC3 cells following *SMG5‐PAQR6* knockdown. (L) *ERV3/1‐ZNF626* is associated with poor prognosis. (M, N) Representative images and histograms of colony formation and transwell migration assays using PC3 cells cocultured with NFs or CAFs. (O, P) Representative images and histograms of colony formation and transwell migration assays using PC3 cells cocultured with CAFs after *ERV3/1‐ZNF626* knockdown. (Q) Schematic diagram of the mechanism of action of chimeric RNAs. ****p < 0.0001, ***p < 0.001, *p < 0.05. FACS, fluorescence‐activated cell sorting; NFs, normal fibroblasts.

The RNA‐seq data of CPGEA was derived from clinical samples rather than cell lines, raising the possibility that some chimeras might be present in the surrounding cells constituting the TME. We used fluorescence‐activated cell sorting (FACS) to isolate cancer cells, CAFs, TAMs, and T cells from Chinese PCa clinical samples (Figure 2A). After verifying the effectiveness of sorting (Figure S4H), we extracted RNA from these cells for further validation. Among the 101 chimeras, 65 were verified in cancer cells, 45 in CAFs, 44 in TAMs, and 27 in T cells (Figure 2B, Figures S5–S8).

We further examined chimeric RNA using existing immortalized cell lines. Since no available CAF cells were currently accessible, we extracted CAFs from a Chinese PCa patient. After validation by Western blot (Figure S9), the CAFs were immortalized [14]. Among the 101 chimeras, 81 were verified in cancer cells, 46 in CAFs, 53 in TAMs, and 59 in T cells (Figures S10–S13). The chimeric RNA profiles of immortalized cell lines differed from those of clinical cell lines (Figure 2C). We believe that tumor heterogeneity is a key factor contributing to this difference. It should be noted that the immortalized cell lines, except for CAFs, were all derived from the Western population. Some observations from these cell lines may need validation once cell lines from Chinese patients are available.

### Functional characterization of chimeric RNAs

To evaluate the protein‐coding potential, we extracted ribosome‐associated RNA from PCa cell lines. 59 chimeras were found bound to ribosomes, indicating that most identified chimeras may be translated (Figure 2D, Figure S14). Different localizations of chimeric RNAs within cells often indicate their functional mechanisms. Nucleoplasm isolation showed 28.4% of chimeric RNAs located in cytoplasm, 9.9% in nucleus, and 61.7% in both (Figure 2D, Figure S15). Lastly, circular RNAs (circRNAs) have been reported to contribute to tumor progression [15]. However, whether chimeric circRNAs exist in PCa remains unreported. Our experiments using RNase R treatment identified 8 out of 101 chimeras as potential circRNAs (Figure 2D, Figure S16).

### Chimeric RNAs can directly influence the tumorigenicity of PCa

We selected 58 chimeras common to both immortalized tumor cells and FACS‐sorted clinical cancer cells to conduct loss‐of‐function study. Among them, eight can be knocked down by more than 50% using siRNAs (Figure S17). A novel chimeric *SLC45A3‐ELK4* (e4e2) caught our attention because another e1e2 isoform was reported to promote PCa progression [16].

We designed forward primer annealing to the first exon of *SLC45A3* and a reverse primer annealing to the last exon of *ELK4*. Touch‐down RT‐PCR and Sanger sequencing confirmed *SLC45A3‐ELK4* (e4e2) comprising the first four exons of *SLC45A3* and the last four exons of *ELK4* (Figure 2E,F). We used a reverse primer annealing to *ELK4* exon5 to perform reverse transcription on DNaseI‐treated RNA (R2). Primers annealing to *SLC45A3* exon4 and intron4 were subsequently used, and further Sanger sequencing supporting that *SLC45A3‐ELK4* (e4e2) is a product of cis‐splicing between adjacent genes (Figure 2G,H). Importantly, knocking down *SLC45A3‐ELK4 (e4e2)* significantly reduced the proliferation and migration of tumor cells (Figure 2I,J), suggesting that chimeric RNAs can directly influence tumorigenicity.

### Chimeric RNAs from tumor cells can alter the phenotype of TME cells

*MPC2‐ADCY10* is upregulated in tumors (Figure S18A) and specifically expressed in PSMA+ cancer cells but does not directly affect PCa cell viability (Figure S18B). However, when M0‐phenotype TAMs were cocultured with PC3 in which *MPC2‐ADCY10* was knocked down, M1‐related markers were upregulated, while M2‐related markers were downregulated (Figure 2K), suggesting that *MPC2‐ADCY10* may mediate communication between tumor cells and TAMs.

CAFs were identified in four subtypes, with only Subtypes I and IV promoting cancer progression, characterized by high levels of CD29 and FAP [17]. *SMG5‐PAQR6* is overexpressed in tumor (Figure S18C) and present in cancer cells, but its silencing did not affect PCa cell viability (Figure S18D). When CAFs were cocultured with PC3 cells in which *SMG5‐PAQR6* was knocked down, CD29 and FAP were both significantly downregulated (Figure 2K), suggesting that this chimeric RNA plays a role in mediating communication between tumor cells and CAFs.

### Chimeric RNAs from TME are involved in the regulation of TME and plasticity

THP‐1 cells were induced to generate M0‐, M1‐, and M2‐phenotype TAMs. After confirming the effectiveness of the induction (Figure S19A), we found 62.1% of TAMs‐related chimeras were upregulated in M2, 20.7% were upregulated in M1, and 17.2% showed comparable levels between M1 and M2 (Figure S19B). Jurkat cells were activated and confirmed by the detection of CD25 and CD69 (Figure S20A). 30.8% T cell‐related chimeras were upregulated in activated Jurkat cells, 23.1% were downregulated, and the remaining chimeras showed no significant change (Figure S20B).

We then extracted normal fibroblasts (NFs) and CAFs from three pairs of tumor and adjacent normal samples. CAF‐related markers were used to confirm the accuracy of the target cells (Figure S21A). We focused on 30 chimeras expressed in both clinical CAFs and immortalized CAFs. Despite interpatient heterogeneity, we identified five chimeras that consistently showed an upward trend in CAFs relative to their NFs, and six chimeras demonstrated a consistently decreasing expression trend (Figure S21B). 7 out of them can be silenced more than 50% in immortalized CAFs (Figure S22A,B). Among them, *ERV3/1‐ZNF626* (e1e2) drew our attention due to its elevated expression in tumor samples and association with poor prognosis (Figure 2L, Figure S22C). Silencing *ERV3/1‐ZNF626* (e1e2) in CAFs inhibited the expression of CAF markers (Figure S22D), suggesting its role in maintaining the CAF phenotype. We further established a co‐culture system. Compared to NFs, CAFs significantly promoted the proliferation and migration of PCa cells (Figure 2M,N). Notably, silencing *ERV3/1‐ZNF626* (e1e2) abolished the promotive effects of CAFs on PCa cells (Figure 2O,P), suggesting that the effects of CAFs on PCa cells are partially dependent on *ERV3/1‐ZNF626* (e1e2).

## CONCLUSION

We validated numerous novel chimeric RNAs, offering potential markers for different cells in PCa, complementing known genes. Notably, we confirmed that these chimeric RNAs can regulate tumor progression through at least three mechanisms (Figure 2Q): (1) influencing tumor growth and motility, (2) contributing to communication between tumors and the microenvironment, and (3) regulating the microenvironment.

## METHODS

Detailed procedures including clinical samples collection, experimental methods, data processing techniques for sequencing data, and bioinformatic and statistical analysis approaches are available in the Supplementary Information. The sequences of primers and siRNAs used in this study are listed in Tables S6–S9.

## AUTHOR CONTRIBUTIONS

**Qiong Wang**: Conceptualization; investigation; writing—original draft; writing—review and editing; funding acquisition. **Shunli Yu**: Investigation; writing—original draft; validation; methodology; data curation. **Jirong Jie**: Investigation; validation; data curation; formal analysis. **Justin Elfman**: Methodology; software; data curation; writing—review and editing. **Zhi Xiong**: Validation; resources. **Sandeep Singh**: Methodology; software. **Samir Lalani**: Methodology; writing—review and editing. **Yiwei Wang**: Methodology. **Kaiwen Li**: Resources. **Bisheng Cheng**: Validation. **Ze Gao**: Validation. **Xu Gao**: Resources; supervision; project administration. **Hui Li**: Conceptualization; investigation; funding acquisition; writing—review and editing; resources; project administration; supervision. **Hai Huang**: Supervision; resources; project administration; funding acquisition; visualization; formal analysis.

## CONFLICT OF INTEREST STATEMENT

The authors declare no conflicts of interest.

## ETHICS STATEMENT

The use of tissues and clinical information in this study was approved (No. SYSEC‐KY‐KS‐2020‐201) by the Sun Yat‐sen University's Committees for Ethical Review of Research Involving Human Subjects. All patients submitted their written informed consents.

## Supporting information


**Figure S1.** Discovery and characterization of chimeric RNAs in TCGA.
**Figure S2.** Gel images of RT‐qPCR products from 301 candidate chimeric RNAs in clinical samples from Sun Yat‐sen Memorial Hospital.
**Figure S3.** Sanger sequencing results of all validated chimeric RNAs.
**Figure S4.** The three representative type chimeric RNAs.
**Figure S5.** Gel images of RT‐PCR products from 101 candidate chimeric RNAs in cancer cells isolated from clinical samples.
**Figure S6.** Gel images of RT‐qPCR products from 101 candidate chimeric RNAs in CAFs isolated from clinical samples.
**Figure S7.** Gel images of RT‐ PCR products from 101 candidates chimeric RNAs in TAMs isolated from clinical samples.
**Figure S8.** Gel images of RT‐ PCR products from 101 candidates chimeric RNAs in T cells isolated from clinical samples.
**Figure S9.** Protein levels of CAF‐related markers PDGFRα, FAP, and α‐SMA.
**Figure S10.** Validation of the 101candidate chimeric RNAs in PCa cell lines.
**Figure S11.** Gel images of RT‐PCR products from 101 candidate chimeric RNAs in the CAF cell line.
**Figure S12.** Gel images of RT‐PCR products from 101 candidate chimeric RNAs in TAM‐related cell lines.
**Figure S13.** Gel images of RT‐PCR products from 101 candidate chimeric RNAs in T cells.
**Figure S14.** Validation of the 101 candidate chimeras in PCa cell lines and their ribosomes.
**Figure S15.** Examination of chimeric RNA distribution in the nucleus and cytoplasm.
**Figure S16.** Gel images of RT‐PCR products from validated cancer cell‐related chimeric RNAs after RNase R treatment.
**Figure S17.** The selection of cancer cell‐related chimeric RNAs.
**Figure S18.** Two tumor cell‐derived chimeric RNAs do not influence the phenotype of tumor cells.
**Figure S19.** Differences in chimeric RNA expression among TAMs with different polarization states.
**Figure S20.** Differences in chimeric RNA expression between Jurkat and activated Jurkat cells.
**Figure S21.** Differences in chimeric RNA expression levels between NFs and CAFs.
**Figure S22.** The selection of CAFs‐related chimeric RNAs.

**Table S1.** The list of chimeric RNAs in TCGA after EricScript prediction.
**Table S2.** The list of chimeric RNAs in CPGEA after EricScript prediction.
**Table S3.** The list of 301 chimeric RNAs which exhibit differential read counts between tumor and adjacent normal tissues.
**Table S4.** Single‐molecule long‐read RNA‐seq samples.
**Table S5.** The number of high‐confidence long‐read support.
**Table S6.** Primer sequences for 301candidate chimeric RNAs.
**Table S7.** Primer sequences used to amplify the full length of *SLC45A3‐ELK4*.
**Table S8.** Primer sequences for TAM‐ and T cell‐related markers.
**Table S9.** The sequences of siRNAs.

## Data Availability

Prostate cancer RNA‐Seq data of TCGA were downloaded at https://portal.gdc.cancer.gov/, while the data for CPGEA are controlled available at the Genome Sequence Archive for Human at http://bigd.big.ac.cn/gsa-human/under the accession number PRJCA001124 (https://ngdc.cncb.ac.cn/gsa-human/browse/HRA000099). Due to ethical and legal restrictions, raw RNA‐seq data from the CPGEA are only available upon request to the data manager of the CPGEA Li Jing (ljing@smmu.edu.cn) and subject to local rules and regulations. This includes submitting a proposal to the management team of CPGEA, where upon approval, analysis needs to be done on a local server with protected access, complying with the Personal Information Protection Law of the People's Republic of China (PIPL) and Regulations on the Administration of Human Genetic Resources of the People's Republic of China. The data and scripts used are saved in GitHub https://github.com/vacation2008/iMETA-2025-126. The Ericscript, Ugrep and Agrep in this study are freely available at https://github.com/databio/ericscript, https://github.com/Genivia/ugrep/, and https://www.tgries.de/agrep/. Supplementary materials (methods, figures, tables, graphical abstract, slides, videos, Chinese translated version, and update materials) can be found in the online DOI or iMeta Science http://www.imeta.science/.
