# Supplementary material for: Profiling chimeric RNA in prostate cancer in Chinese cohorts reveals similarities and differences compared to Western populations
Source: Imeta. 2025 Mar 13;4(2):e70014. doi: 10.1002/imt2.70014 (PMC11995180; doi:10.1002/imt2.70014)
Supplement: Supplementary file 1 — Figure S1. Discovery and characterization of chimeric RNAs in TCGA. Figure S2. Gel images of RT‐qPCR products from 301 candidate chimeric RNAs in clinical samples from Sun Yat‐sen Memorial Hospital. Figure S3. Sanger sequencing results of all validated chimeric RNAs. Figure S4. The three representative type chimeric RNAs. Figure S5. Gel images of RT‐PCR products from 101 candidate chimeric RNAs in cancer cells isolated from clinical samples. Figure S6. Gel images of RT‐qPCR products from 101 candidate chimeric RNAs in CAFs isolated from clinical samples. Figure S7. Gel images of RT‐ PCR products from 101 candidates chimeric RNAs in TAMs isolated from clinical samples. Figure S8. Gel images of RT‐ PCR products from 101 candidates chimeric RNAs in T cells isolated from clinical samples. Figure S9. Protein levels of CAF‐related markers PDGFRα, FAP, and α‐SMA. Figure S10. Validation of the 101candidate chimeric RNAs in PCa cell lines. Figure S11. Gel images of RT‐PCR products from 101 candidate chimeric RNAs in the CAF cell line. Figure S12. Gel images of RT‐PCR products from 101 candidate chimeric RNAs in TAM‐related cell lines. Figure S13. Gel images of RT‐PCR products from 101 candidate chimeric RNAs in T cells. Figure S14. Validation of the 101 candidate chimeras in PCa cell lines and their ribosomes. Figure S15. Examination of chimeric RNA distribution in the nucleus and cytoplasm. Figure S16. Gel images of RT‐PCR products from validated cancer cell‐related chimeric RNAs after RNase R treatment. Figure S17. The selection of cancer cell‐related chimeric RNAs. Figure S18. Two tumor cell‐derived chimeric RNAs do not influence the phenotype of tumor cells. Figure S19. Differences in chimeric RNA expression among TAMs with different polarization states. Figure S20. Differences in chimeric RNA expression between Jurkat and activated Jurkat cells. Figure S21. Differences in chimeric RNA expression levels between NFs and CAFs. Figure S22. The selection of CAFs‐related chim [file IMT2-4-e70014-s002.docx]

**Supporting information to**

# **Profiling chimeric RNA in prostate cancer in Chinese cohorts reveals similarities and differences compared to western populations**

**Running title:** Chimeric RNAs in Chinese PCa population

Qiong Wang^1,2,3#^, Shunli Yu^3,4#^, Jirong Jie^1#^, Justin Elfman^2^, Zhi Xiong^3,4^, Sandeep Singh^2^, Samir Lalani^2^, Yiwei Wang^2^, Kaiwen Li^3,4^, Bisheng Cheng^1^, Ze Gao^3,4^, Xu Gao^5^*, Hui Li^2^*, Hai Huang^3,4,6^*

^1^Department of Urology, Nanfang Hospital, Southern Medical University, Guangzhou 510515, China

^2^Department of Pathology, School of Medicine, University of Virginia, Charlottesville VA 22908, USA

^3^Department of Urology, Sun Yat-sen Memorial Hospital, Sun Yat-sen University, Guangzhou 510120, China

^4^Guangdong Provincial Key Laboratory of Malignant Tumor Epigenetics and Gene Regulation, Sun Yat-Sen Memorial Hospital, Sun Yat-Sen University, Guangzhou 510120, China

^5^Department of Urology, Changhai Hospital, Naval Medical University, Shanghai 200433, China

^6^Department of Urology, The Sixth Affiliated Hospital of Guangzhou Medical University, Qingyuan People’s Hospital, Qingyuan 511518, China

^*^Correspondence: huangh9@mail.sysu.edu.cn (Hai Huang); hl9r@virginia.edu (Hui Li); gaoxu.changhai@foxmail.com (Xu Gao).

**Supplementary methods**

**Bioinformatics**

Prostate cancer RNA-Seq data of The Cancer Genome Atlas (TCGA) were downloaded at <https://portal.gdc.cancer.gov/>, while the data for Chinese populations are available at the Genome Sequence Archive for Human at <http://bigd.big.ac.cn/gsa-human/>. We used EricScript software (version 0.5.5b) [1, 2] with default parameters and the hg38 reference genome to predict chimeric RNAs. We discarded chimeric RNAs with an EricScore of less than 0.6 and filtered out false positive events using BLAT filtering. The University of California Santa Cruz (UCSC) Genome Browser (<http://genome.ucsc.edu>) was utilized to confirm the authenticity of candidate chimeras. KEGG pathway analysis for the parental genes of chimeric RNAs was performed using the SangerBox tools, available at http://sangerbox.com/. Additionally, Agrep software (<https://www.tgries.de/agrep>) was employed to calculate the read counts of target chimeras or parental genes in the CPGEA.

Samples utilized for *in silico* validation are listed in Table S4. Ugrep (<https://github.com/Genivia/ugrep/>) was performed as previously described [3], using 14 bp from either side of the chimeric junction. Each read was aligned to Hg38 using BLAT to confirm alignment to the correct locus. Up to five reads from each chimeric RNA with on-target single-molecule long read support were aligned to hg38 with BLAT and manually assessed against the Comprehensive Gene Annotation Set from GENCODE Version 44 (Ensembl 110). The results are listed in Table S5.

**Clinical samples**

Fresh tumor and adjacent normal tissues from 32 PCa patients were collected at Sun Yat-sen Memorial Hospital to validate candidate chimeras. Additionally, another 20 fresh clinical samples were used for fluorescence-activated cell sorting (FACS) to isolate tumor cells, cancer-associated fibroblasts (CAFs), tumor-associated macrophages (TAMs), and T cells, with 5 samples from each cell type.

**RNA extraction, RT-qPCR, touch-down PCR, agarose electrophoresis and Sanger sequencing**

Total RNA was extracted from the cells using TRIzol reagent (15596026, Invitrogen, United States), following the method previously described [4]. The complementary DNA was synthesized with random hexamer primer using the Verso cDNA Synthesis Kit (AB1453A, Thermo Fisher Scientific, United States). Quantitative real-time PCR (RT-qPCR) was carried out on ABI StepOne Plus real-time PCR system (4376600, Applied Biosystems, United States). Primers for the 301 chimeric RNAs were listed in Table S6. Touch-down PCR (TD-PCR) was carried out using the Platinum Taq High Fidelity Ki (11304011, Invitrogen, United States). Primers for amplifying the full length of *e4e2* *SLC45A3-ELK4* were listed in Table S7. Primers for T cells and TAMs-related markers were listed in Table S8.

2% agarose gel was made for separating 100-300bp DNA products, and 1% agarose gel for longer DNA products. The target bands were cut for Sanger sequencing at RuiBiotech.

**Single-cell isolation and construction of an immortalized CAFs cell line**

Fresh tissues were isolated from clinical PCa patients and rinsed with precooled PBS until no visible blood remained. The charred regions from ultrasonic scalpel and adipose tissue were removed. The remaining tissues were cut into pieces and digested with 0.1% Liberase (1020763; Roche; Switzerland) under continuous oscillation at 37°C for 40 minutes. The suspension was then filtered through a 200-μm nylon filter mesh to obtain a single-cell suspension. The resulting cell population was a mixture of all types of cells, which were used in two ways: 1) co-cultured with the following fluorescent conjugated antibodies: PE anti-human CD14 (325605, Biolegend, United States), Alexa Fluor 488 anti-human PSMA (342505, Biolegend, United States), Brilliant Violet 421™ anti-human CD3 (317343, Biolegend, United States), and PE anti-human PDGFRα (323505; Biolegend, United States) following by FACS to isolate TAMs, tumor cells, T cells and CAFs; 2) constructed immortalized CAFs.

The isolated CAFs that were positive for PDGFRα were seeded in 10-cm cell culture plates and cultured at 37°C in a 5% humidified carbon dioxide environment. Following western blot validation for α-SMA, FAP, and PDGFRα, the CAFs were sent to Applied Biological Materials (ABM) for immortalization [5].

**Protein isolation and Western blotting**

Protein isolation and western blot analysis were conducted as previously described [1]. The proteins in the cell samples were harvested using RIPA lysis buffer (P0013B , Beyotime, China) and separated by 10% sodium dodecyl sulfate polyacrylamide gel electrophoresis. The proteins were then transferred to polyvinylidene fluoride membranes and incubated with the following primary antibodies at 4°C for 16h: PDGFRα (ab32570; Abcam; United Kingdom), GAPDH (GB11002; Servicebio; China), FAP (A6349; Abclonal; China), α-SMA (A17910; Abclonal; China), CD29 (12594-1-AP; Proteintech; United States).

The membranes were then incubated with the secondary antibody at room temperature for 1 h. The protein band signals were visualized using Immobilon Western Chemiluminescent HRP Substrate (WBKLS0500, Merck Millipore, Germany).

**Cell culture**

Jurkat, THP-1, LNCaP, C4-2, PC3, DU145, and 22Rv1 were purchased from ATCC (American Type Culture Collection). Primary NFs, primary CAFs, and immortalized CAFs were obtained from clinical samples according to the procedure above. The cells mentioned were cultured in RPMI 1640 (Jurkat, THP-1, LNCaP, C4-2, PC3, and 22Rv1) or Dulbecco’s Modified Eagle’s Medium (primary NFs, primary CAFs, immortalized CAFs, DU145) (11965092, Gibco, United States), supplemented with 10% fetal bovine serum (A5256701, Gibco, United States) and 1% pen/strep (15140122, Gibco, United States). Cells were maintained at 5% CO2 in a 37C humidified incubator.

**The activation of Jurkat and the polarization of THP-1**

Jurkat cells were stimulated with CD3/CD28 Dynabeads at a 1:1 ratio (11161D, Gibco, United States). After 24 hours of stimulation, the activation of the Jurkat cells was confirmed by RT-qPCR.

THP-1 cells were cultured with 150nM PMA (70-CS0001, Lianke Bio, China) for 24 hours to differentiate into M0-type TAMs. M0 macrophages were then cultured in serum-free RPMI 1640 medium containing 20ng/ml interferon-gamma (IFN-γ) (Z02915-10, GenScript, China) and 10pg/ml lipopolysaccharide (LPS) (L861706, Macklin, China) for 24 hours to induce M1 macrophages. Similarly, M2 macrophages were obtained by exposing M0 macrophages to serum-free medium supplemented with 20ng/ml interleukin-4 (IL-4) (PRP2017, Abbkine, China) and 20ng/ml interleukin-13 (IL-13) (PRP100108, Abbkine, China) for 24 hours. RT-qPCR was used to confirm the differentiation statuses of M0, M1, and M2 macrophages.

**siRNA transfection**

RNA interference (siRNA) oligonucleotides targeting selected chimeric RNAs were purchased from GenePharma (Shanghai, China). The siRNA sequences are listed in Table S9. siRNA transfections were performed using 75nM siRNA mixed with 3µL/mL Lipofectamine RNAiMAX (13778150, Thermo Fisher Scientific, United States) and incubated for 48 hours to allow for RNA isolation and functional cell testing.

**Colony formation assay and migration assay**

The colony formation assay was performed to detect cell viability. PCa cells (1000 DU145 cells or 1500 PC3 cells per well) were seeded into 6-well plates and cultured for 10 days to calculate the number of colonies.

A migration assay was performed by filling the bottom well of the cell culture insert (353097, Corning, United States) with DMEM or RPMI 1640 medium containing 10% FBS. The insert wells were covered with polyethylene terephthalate (PET) membranes with 8-µm pores, and 40,000 cells/well in serum-free DMEM or RPMI 1640 were added to the top culture insert. The cell culture insert chamber was incubated at 37°C, allowing 10 hours for DU145 cells and 24 hours for PC3 cells, to enable cell migration through the membrane into the bottom chamber. The membranes were stained with crystal violet, and cells that migrated to the bottom chamber were counted using an Olympus IX71 inverted microscope (Olympus, Japan).

**Statistical analyses**

Quantitative results in this study were reported as the mean ± standard deviation (SD) and assessed with an ANOVA test followed by Tukey's post-hoc test for multiple comparisons (GraphPad, La Jolla, CA, USA). Paired samples in TCGA and CPGEA were analyzed using paired Student’s t-test. The Kaplan-Meier method was used to describe BCR-free survival, and a *p*-value < 0.05 was considered statistically significant after the Log-rank test. *P*-values were labeled in figures as follows: **p* < 0.05, ***p* < 0.01, ****p* < 0.001, *****p* < 0.0001.

**Reference**

1. Wang, Qiong, Bisheng Cheng, Sandeep Singh, Yiran Tao, Zhongqiu Xie, Fujun Qin, Xinrui Shi, et al. 2024. “A protein-encoding CCDC7 circular RNA inhibits the progression of prostate cancer by up-regulating FLRT3.” *NPJ Precision Oncology* 8: 11. <https://doi.org/10.1038/s41698-024-00503-2>

2. Singh, Sandeep, Hui Li. 2021. “Comparative study of bioinformatic tools for the identification of chimeric RNAs from RNA Sequencing.” *RNA Biology* 18: 254−267. <https://doi.org/10.1080/15476286.2021.1940047>

3. Elfman, Justin, Lynette Goins, Tessa Heller, Sandeep Singh, Yuh-Hwa Wang, Hui Li. 2024. “Discovery of a polymorphic gene fusion via bottom-up chimeric RNA prediction.” *Nucleic Acids Research* 52: 4409−4421. <https://doi.org/10.1093/nar/gkae258>

4. Wang, Qiong, Wanhua Wu, Ze Gao, Kaiwen Li, Shirong Peng, Huiyang Fan, Zhongqiu Xie, Zhenghui Guo, Hai Huang. 2021. “GADD45B Is a Potential Diagnostic and Therapeutic Target Gene in Chemotherapy-Resistant Prostate Cancer.” *Frontiers in Cell and Developmental Biology* 9: 716501. <https://doi.org/10.3389/fcell.2021.716501>

5. Xiong, Zhi, Shun-Li Yu, Zhao-Xiang Xie, Rui-Lin Zhuang, Shi-Rong Peng, Qiong Wang, Ze Gao, et al. 2024. “Cancer-associated fibroblasts promote enzalutamide resistance and PD-L1 expression in prostate cancer through CCL5-CCR5 paracrine axis.” *iScience* 27: 109674. <https://doi.org/10.1016/j.isci.2024.109674>

**Supplementary figures**

**Figure S1** **Discovery and characterization of chimeric RNAs in TCGA.** (A-B) BCR-free survival analyses based on the number of inter-chromosomal (A) and intra-chromosomal (B) chimeric RNAs in CPGEA and TCGA database. (C-D) No significant difference in read-through chimeras was found between tumor and adjacent tissues in CPGEA, while TCGA showed a significant increase in tumors compared to adjacent normal samples. (E) After filtering out 'M/M' fusions and those with a recurrence of less than four, 1,351 chimeric RNAs were identified using UCSC Genome Browser in CPGEA, and 384 in TCGA. (F) The pipeline for validating chimeric RNAs in CPGEA. **p* < 0.05.

**Figure S2** **Gel images of RT-qPCR products from 301 candidate chimeric RNAs in clinical samples from Sun Yat-sen memorial hospital.** Validated chimeras are highlighted in red after Sanger sequencing.

**Figure S3** **Sanger sequencing results of all validated chimeric RNAs.** Junction sites are marked with blue lines.

**Figure S4** **The three representative Type chimeric RNAs.** (A) Overlap between the experimental validated and *in silico* validated chimeras. (B-C) Representative Type I (*MBD1-CFAP53*) chimeric RNA was highly expressed in tumor samples and asscoiated with P53 pathway related genes. (D-E) Representative Type II (*RAB3B-NRD1*) chimeric RNA was highly expressed in tumor samples and asscoiated with DNA damage related genes. (F-G) Representative Type III (*DUS4L-BCAP29*) chimeric RNA was highly expressed in tumor samples and asscoiated with AR pathway related genes. (H) Verification of the effectiveness of PSMA, PDGFRα, CD14, and CD3 antibodies for FACS.

**Figure S5** **Gel images of RT-PCR products from 101 candidate chimeric RNAs in cancer cells isolated from clinical samples.** Chimeras detected in clinical cancer cells are highlighted in red.

**Figure S6** **Gel images of RT-qPCR products from 101 candidate chimeric RNAs in CAFs isolated from clinical samples.** Chimeras detected in clinical CAFs are highlighted in red.

**Figure S7** **Gel images of RT- PCR products from 101 candidates chimeric RNAs in TAMs isolated from clinical samples.** Chimeras detected in clinical TAMs are highlighted in red.

**Figure S8** **Gel images of RT- PCR products from 101 candidates chimeric RNAs in T cells isolated from clinical samples.** Chimeras detected in clinical T cells are highlighted in red.

**Figure S9** **Protein levels of CAF-related markers PDGFRα, FAP, and α-SMA.** CAFs were extracted from a Chinese PCa patient using FACS.

**Figure S10** **Validation of the 101 candidate chimeric RNAs in PCa cell lines.** Mixed RNAs from LNCap, C4-2, PC3, DU145, and 22Rv1 were used. Validated chimeras are highlighted in red after Sanger sequencing.

**Figure S11** **Gel images of RT-PCR products from 101 candidate chimeric RNAs in the CAF cell line.** Validated chimeras are highlighted in red after Sanger sequencing.

**Figure S12** **Gel images of RT-PCR products from 101 candidate chimeric RNAs in TAM-related cell lines.** Mixed RNAs from THP-1, M0, M1, and M2 cells were used. Validated chimeras are highlighted in red after Sanger sequencing.

**Figure S13** **Gel images of RT-PCR products from 101 candidate chimeric RNAs in T cells.** Mixed RNAs from Jurkat and activated Jurkat cells were used. Validated chimeras are highlighted in red after Sanger sequencing.

**Figure S14** **Validation of the 101 candidate chimeras in PCa cell lines and their ribosomes.** (A) Gel images of RT-PCR products from 101 candidate chimeric RNAs in PCa cell lines (LNCaP, C4-2, PC3, DU145, and 22Rv1) and their derived ribosomes. The left side represents total RNAs from cell lines, while the right side represents ribosomal enriched RNAs. (B) Gel images of RT-qPCR products from the rest of candidate chimeric RNAs in ribosomes enriched RNAs. Chimeras detected in both the cell line and ribosome are marked in blue, those detected only in the cell line but not in the ribosome are marked in red, and those detected in neither are marked in black.

**Figure S15** **Examination of chimeric RNA distribution in the nucleus and cytoplasm.** Mixed RNAs from LNCap, C4-2, PC3, DU145, and 22Rv1 cells were used. Chimeras detected in both the nucleus and cytoplasm are marked in blue. Chimeras detected only in the nucleus but not in the cytoplasm are marked in black. Chimeras detected only in the cytoplasm but not in the nucleus are marked in red.

**Figure S16** **Gel images of RT-PCR products from validated cancer cell-related chimeric RNAs after RNase R treatment.** Chimeras resistant to RNase R are marked in red.

**Figure S17** **The selection of cancer cell-related chimeric RNAs.** (A) The pipeline for selecting cancer cell-related chimeric RNAs. (B) RT-qPCR analysis of knockdown efficiency for selected chimeric RNAs in PC3, C4-2, and DU145 cells after specific siRNA transfection. ****p* < 0.001, ***p* < 0.01, **p* < 0.05.

**Figure S18** **Two tumor cell-derived chimeric RNAs do not influence the phenotype of tumor cells.** (A) *MPC2-ADCY10* is upregulated in tumor samples. (B) Colony formation assay ssessing cell viability in DU145 and PC3 cell lines after knockdown of *MPC2-ADCY10*. (C) *SMG5-PAQR6* is upregulated in tumor samples. (D) Colony formation assay assessing cell viability in DU145 and PC3 cell lines after knockdown of *SMG5-PAQR6*. *****p* < 0.0001.

**Figure S19 Differences in chimeric RNA expression among TAMs with different polarization states.** (A) Monocyte-related marker CD11b, M1-phenotype-related markers CD86 and TNF-α, and M2-phenotype-related markers CD206 and ARG1 were detected by RT-qPCR to confirm the effectiveness of THP-1 induction. (B) RT-qPCR analysis of changes in TAM-related chimeric RNAs after THP-1 cells were induced into M0, M1, and M2 phenotypes. *****p* < 0.0001, ****p* < 0.001, ***p* < 0.01, **p* < 0.05.

**Figure S20** **Differences in chimeric RNA expression between Jurkat and activated Jurkat cells.** (A) Activated T cell-related markers CD25 and CD69 were detected by RT-qPCR to confirm the effectiveness of Jurkat cell activation. (B) Quantitative analysis of changes in T cell-related chimeric RNAs after Jurkat activation. *****p* < 0.0001, **p* < 0.01, **p* < 0.05.

**Figure S21** **Differences in chimeric RNA expression levels between NFs and CAFs.** (A) Protein levels of CAF-related markers PDGFRα, FAP, and α-SMA were detected by Western blot, confirming the successful extraction of CAFs from three pairs of clinical PCa samples. (B) RT-qPCR analysis of changes in CAF-related chimeric RNAs between NFs and CAFs. ****p* < 0.001, ***p* < 0.01, **p* < 0.05.

 **Figure S22 The selection of CAFs-related chimeric RNAs.** (A) The pipeline for selecting CAF-related chimeric RNAs. (B) RT-qPCR analysis of knockdown efficiency for selected chimeric RNAs in CAF cells after specific siRNA transfection. (C) *ERV3/1-ZNF626* is upregulated in tumor samples. (D) Western blot analysis of changes in CAF-related markers after ERV3/1-ZNF626 knockdown in CAF cell lines. *****p* < 0.0001, ****p* < 0.001, ***p* < 0.01, **p* < 0.05.
